# The classical progesterone receptor mediates the rapid reduction of fallopian tube ciliary beat frequency by progesterone

**DOI:** 10.1186/1477-7827-11-33

**Published:** 2013-05-08

**Authors:** Anna Bylander, Karin Lind, Mattias Goksör, Håkan Billig, DG Joakim Larsson

**Affiliations:** 1 Institute of Neuroscience and Physiology, the Sahlgrenska Academy , University of Gothenburg, SE-405 30 Göteborg, Sweden; 2 Department of Physics, University of Gothenburg, SE-412 96 Göteborg, Sweden; 3 Department of Infectious Diseases, Institute of Biomedicine, the Sahlgrenska academy, University of Gothenburg, Guldhedsgatan 10, SE-413 46 Göteborg, Sweden

**Keywords:** Progesterone, Fallopian tube, Ciliated cell, Rapid response, Nuclear progesterone receptor

## Abstract

**Background:**

The transport of gametes as well as the zygote is facilitated by motile cilia lining the inside of the fallopian tube. Progesterone reduces the ciliary beat frequency within 30 minutes in both cows and mice. This rapid reduction suggest the involvement of a non-genomic signaling mechanism, although it is not known which receptors that are involved. Here we investigated the possible involvement of the classical progesterone receptor in this process.

**Method:**

The ciliary beat frequency of mice fallopian tube was measured *ex vivo* using an inverted bright field microscope and a high speed camera. The effects of the agonists progesterone and promegestone and an antagonist, mifeprestone, were investigated in wildtype mice. The effect of progesterone was also investigated in mice lacking the classical progesterone receptor.

**Results:**

Progesterone, as well as the more specific PR agonist promegestone, significantly reduced the CBF at concentrations of 10–100 nanomolar within 10–30 minutes. In the absence of progesterone, the PR antagonist mifeprestone had no effect on the ciliary beat frequency at a concentration of 1 micromolar. When ciliated cells were pre-incubated with 1 micromolar mifeprestone, addition of progesterone did not reduce the ciliary beat frequency. Accordingly, in ciliated cells from mice not expressing the classical progesterone receptor, exposure to 100 nanomolar progesterone did not reduce the ciliary beat frequency.

**Conclusions:**

This is the first study to provide comprehensive evidence that the classical progesterone receptor mediates the rapid reduction of the tubal ciliary beat frequency by progesterone.

## Background

A major function of the mammalian fallopian tube is to regulate the speed of the egg transport [1]. In addition, the fallopian tube maintains viability and controls the ascent of spermatozoa to the site of fertilization and delivers the fertilized embryo to the uterus [2,3]. The transport of gametes and embryos is aided both by muscular contractions as well as movement of ciliated cells, where the ciliary propulsion seem to be most important [4,5]. It has been proposed that progesterone regulates ciliary beat frequency (CBF) in several mammalian species, and both classical genomic effects and more rapid responses, likely involving some other means of signaling, have been reported [6,7]. In guinea pigs injected *in vivo* with progesterone, the beat frequency of the fimbria of the fallopian tube were decreased 1.5 days after the onset of treatment [8]. In humans, a reduction of CBF by 40-50% was observed 24 hour after treatment with 10 μM progesterone *in vitro*, suggesting a direct effect of the hormone [6]. In cows, progesterone caused a rapid reduction of CBF within 30 minutes after exposure to 20 μM progesterone [7]. These *in vitro* studies have used rather high concentrations of the steroid, and it was therefore not clear if physiological levels of progesterone could evoke similar effects. Recent studies in our lab on mice have, however, demonstrated that the CBF is reduced within 30 minutes by 100 nM progesterone [9]. This concentration can be found in blood serum of cycling women, whereas in mice, the highest serum levels of progesterone during the reproductive cycle appear to be somewhat lower, varying between 25 nM in early estrus and 50 nM in metestrus and diestrus, [6,10]. This quite rapid response suggests that progesterone might regulate CBF in the fallopian tube via another pathway than the classical genomic mechanism. We have speculated that secreted progesterone from the travelling cumulus complex could act as a local beacon to the cells of the fallopian tube, thereby communicating its detailed position [9,11,12].

Previous studies indicate that at least two different classes of progesterone receptors are expressed in the ciliated cells of mice and humans, i.e. membrane PRs, (mPRs) [11,12] together with the classical PR (PR) [13], and both types of receptors are therefore candidates for mediating rapid effects of progesterone in the fallopian tube [14]. The mPRs, (mPRα, β, and γ), were initially described by Zhu et al. [15,16]. After stimulation with progesterone mPRs seems to be involved in processes such as oocyte maturation [17,18] and has also been proposed to regulate sperm motility through interactions with G-proteins [19]. Apart from the well characterized genomic mechanism of progesterone action, the classical PR mediates rapid non-genomic signaling in several cell types such as immortalized breast cancer cells [20-22]. These non-genomic effects seem to be mediated by the PR-B isoform [23,24]. Evidence suggest that PR do not only transfer between the cytoplasmic and nuclear compartments to activate transcription, but also contains an SH3 domain interaction motif that mediates the non-genomic actions of progesterone through activation of the Raf-MAPK signaling pathway [23,25]. This suggests that PR has dual functions as a nuclear transcription factor and as an activator of cell signaling molecules [24,26]. In the fallopian tube, PR has been proposed to have an unusual localization on the cilia rather than in the nucleus [13], supporting role in regulating ciliary function independent of gene transcription.

It is important to reveal the mechanisms regulating transport of gametes and ciliary motility since dysfunction of the fallopian tube and cilia can lead to ectopic pregnancies and other forms of infertility [27-31]. Furthermore, interference with gamete transport is a possible target for novel contraceptive treatment strategies. In a previous paper we developed a method to study CBF in mice and found that progesterone caused a reduction in CBF within 30 minutes after administration [9]. Currently, there are few tools available to specifically manipulate mPRs (specific agonists/antagonist, knock-out mice etc.), however such tools are available to pursue the role of the PR. The aim of this study was therefore to investigate the possible involvement of the PR in the rapid regulation of CBF by progesterone.

## Methods

### Animals

The experiments were performed in principal as described by Bylander *et al.*[9] with some minor improvements. Fallopian tubes were obtained from immature (3.5-5 weeks-old) female C57BL/6 mice from Charles River, Kisslegg, Germany and from PR knock-out mice, (PRlacZ), kindly provided by the group of Dr Michael Schumacher from University Paris-Sud, France. The animals were kept under a 12:12-h light–dark schedule at 21 ± 2°C with ad libitum access to chow and water. Before the start of the experiment, the animals were allowed to acclimate to the animal facilities for ≥ 5 days. All experiments were approved by the local animal ethics committee in, Gothenburg Sweden (246/2007, 392/2008 and 50/2011) to DGJL.

### Genotyping of PRlacZ mice

In the PRlacZ mice the lacZ reporter has been knocked into exon 1 of the murine PR gene. With a strategy to insert the lacZ reporter 120 amino acids downstream of (and in-frame to) the initiating codon for the PR-B isoforms a short region of the N-terminal domain, containing the initiating codon for the PR-A form, was deleted [32]. The mice received in our lab, i.e. three heterozygote females and two heterozygote males PR(+/−) and one male knock-out PR (−/−) constituted three breeding pairs. The offspring were genotyped by genomic DNA isolated from their ears, analysed by touchdown polymerase chain reaction (PCR). The following PR-specific primers were used: P1: 5’ tag aca gtg tct tag act cgt tgt tg 3’, P3: 5’ gat ggg cac atg gat gaa atc 3’ and PRlacZ 5’ ctt cac cca ccg gta cct tac gtc tc. Only female knock-out mice (PR−/−) were used for the experiments.

### Tissue collection and sample preparation

The mice were anesthetized by inhalation of isoflourane and then killed by inhalation of carbon dioxide followed by dissection. Both fallopian tubes were immediately isolated and washed in PBS (Phosphate Buffered Saline, pH 7.0). The broad ligament, fat and blood vessels were removed and the segments reaching from the ampullary to the infundibulum region of the fallopian tube were transferred to petri-dishes containing 37°C pre-warmed F-10 Nutrient Mixture (Ham, 1X) plus 10% fetal bovine serum (Invitrogen, Sweden). After an additional washing step, the tube-like segments were longitudinally opened and chopped into smaller pieces and retransferred to fresh medium and kept in an incubator (37°C, 5% CO_2_ and 100% humidity) until measurements the following day.

The CBF recordings were performed at a temperature of about 37°C. Pieces of tissue were placed in petri dishes (35 mm) with glass bottom microwells (14 mm) (MatTek Corporation, Ashland, USA) containing 1 ml of pre-warmed G-MOPS™(Vitrolife, Gothenburg, Sweden). This is a medium specially developed for handling and manipulation of oocytes and embryos in under normal air. When a sample with beating cilia was located, the piece of tissue was secured to the bottom of the dish by a sterilized needle (TransferTip®, iD 15 μM, oD 20 μm, Eppendorf AG, Hamburg, Germany) connected to a motorized microinjection system (TransferMan, Eppendorf). Only cells at the perimeter of the tissue were used for analysis and on each sample, the CBFs of one or two different cells were measured. A total number of 124 cells and 48 mice were used in all the experiments.

### Detection system

For the CBF measurements we used a method developed in a previously study by Bylander et al. [9]. In short, an inverted bright field microscope (Nikon TE-300, Nikon Instruments, Inc, New York) equipped with a 100× oil immersion objective ( NA1.3) was used. To allow measurements at a temperature of 37°C, the microscope objective was heated by an adjustable heater loop attached to the objective and regulated by a controller unit (Bioptechs, Butler, Pennsylvania). Images were acquired with a 12 bit high speed camera (Prosilica EC1020, Prosilica Inc, Burnaby, Canada). A region of interest of approximately 50×50 pixels was recorded with a speed of 100 frames per second. From the recorded images the spatial positions of cilia attached to a single cell were determined by a center-of-mass algorithm in LabView (National Instruments, Austin, TX). The center of mass for an image of ciliated cells is a point in space at which the cilia´s whole mass can be considered to be concentrated to and facilitate calculating the ciliary movement. Finally, with a fast Fourier transform (FFT) in which the recorded ciliary movements are decomposed into components of different frequencies the distribution of beat frequencies present was calculated.

## Drug exposure

### Agonists

Progesterone acts as an agonist both to the mPRs and the PR. Promegestone (R5020) is similarly an agonist to the PR, but have low affinity to mPRs [33-35]). Progesterone (>99% purity, Sigma-Aldrich, St Louis, MO) and promegestone (R5020, >99% purity, Sigma-Aldrich, St Louis, MO) was first dissolved in ethanol and then through further dilutions steps into G-MOPS™. To add a drug to the cells, half of the medium in the dish was replaced with an equal volume of medium containing ethanol and drug, resulting in a final exposure concentration of 0.01% (v/v) ethanol and a series of concentrations of progesterone ranging from 10 nM up to 100 nM and a concentration of 100 nM R5020. Control cells were exposed to ethanol (0.01% (v/v)) only.

A baseline frequency for each cell was established based on repeated measurements during 10 minutes, after which the drug was added together with medium. The CBF was then recorded every 5 minutes for the following 60 minutes. We knew from previous studies that 100 nM progesterone affects the CBF within 30 minutes in both cow and mice [7,9], so to allow a response to develop, the average CBF of the last 30 minutes of the recording period was calculated and statistically compared with the baseline frequency for each cell to determine the effect of added drug.

### Antagonist

To investigate whether or not the CBF reduction by progesterone could be blocked by an antagonist to the PR we used mifepristone also referred to as RU486 (Sigma-Aldrich, St Louis, MO). Mifepristone has very low affinity to the mPRs [34,35]. First, a study was performed to investigate if 1 μM of RU486 alone affected the CBF. After a baseline frequency was established, RU486 was added to a final concentration of 1 μM in G-mops™ medium and the CBF was measured during 90 minutes. The time period of 90 minutes rather than our standard 60 minute protocol was used here since we were interested in comparing the results from these particular experiments with a second experiment where cells were pretreated with RU486 for 30 minutes (1 μM) after which medium with both RU486 and progesterone was added to final concentrations of 1 μM and 100 nM, respectively, and the CBF measured during an additional 60 minutes. A tenfold higher concentration of RU486 compared with progesterone was used in order to provide suitable conditions for blocking the action of progesterone. In both experiments, control cells as well as cells treated with RU486/progesterone were exposed to ethanol (0.01% (v/v).

### PRlacZ mice

Ciliated cells from PRlacZ mice were exposed to progesterone at a concentration of 100 nM, known to affect the CBF in wildtype mice ([9]; present study). When a baseline frequency was established, progesterone was added and the CBF was measured during 60 minutes. The control cells were exposed to ethanol only (0.01% (v/v)).

### Statistics

The mean baseline CBF for all wildtype and PRLacZ mice used in the experiment was 27.3 and 20.6 Hz respectively. Furthermore, as the baseline CBF for different mice and even different cells from the same mouse vary considerably, the CBF after treatment the CBF for each cell was always normalized against its own baseline CBF. Thus, for each ciliated cell, regardless of the type of treatment, the difference in frequencies (Δ frequency) before and after treatment was calculated as the difference between the mean CBF during the last 30 minutes of the measurement and the baseline CBF. In some cases the frequency was measured in parallel on two cells on a piece of tissue, and if so the average Δ frequency of the two cell replicates was used for further calculations. For each mouse the difference in CBF between tissue exposed to a drug and the control tissue was calculated. This paired approach was used for graphical illustration of the changes over time in response to treatment with the drugs. In order to statistically assess the dose-dependent effect of progesterone, one way Anova followed by a Dunnett’s post hoc test was applied. For the four experiments with R5020, RU486, and progesterone-treatment of PRlacZ mice we were able to obtain results from both exposed and control tissue for each of the mice included. Thus, a paired two-sided *t*-test was used when analyzing these experiments, providing additional statistical control for any additional variability due to variations between individual donor mice.

## Results

**Figure 1:**
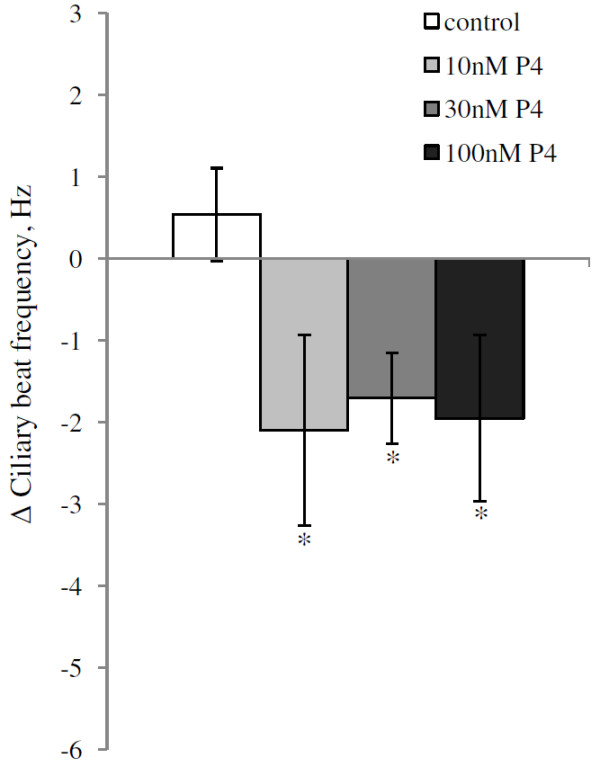
**Average change in ciliary beat frequency (CBF) for ciliated cells in fallopian tube of mice after treatment with progesterone.** The change in CBF is the difference between the average CBF during 30–60 minutes after addition of progesterone and the average baseline CBF. The CBFs are presented as mean ± SEM. Control cells were exposed to ethanol (EtOH) only. The significance is shown as * P < 0.05. The number of cells used in the experiment were: control = 12, 10 nM = 5, 30 nM = 8 and 100 nM = 10 derived from a total of 13 mice. When several cells were obtained and used from the same mouse, the cells were distributed to different treatment groups.

**Figure 2:**
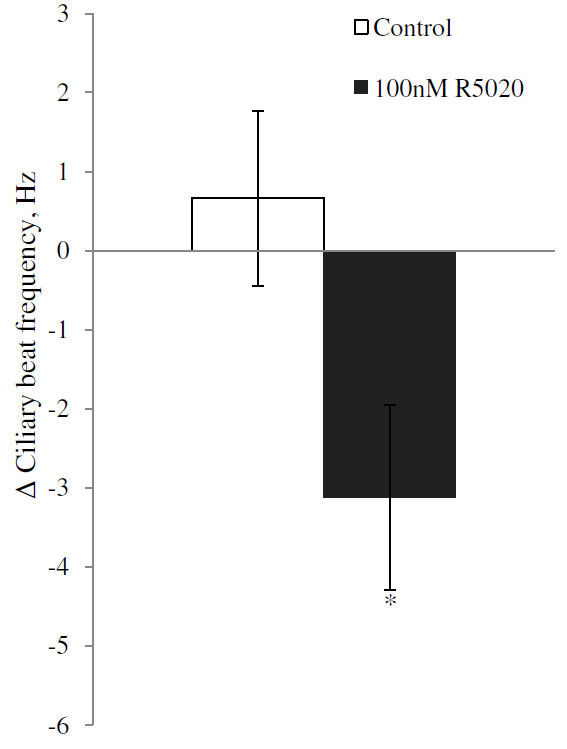
**Average change in ciliary beat frequency (CBF) for ciliated cells in fallopian tube of mice after treatment with 100 nM R5020.** The change in CBF is the difference between the average CBF during 30–60 minutes after addition of R5020 and the average CBF for the baseline period. CBF is presented as mean ± SEM. Cells exposed to ethanol (EtOH) are used as a control. The significance is shown as * P < 0.05. The number of cells used were 8 in each group from in total 8 animals. For each animal, one cell was allocated to each treatment group.

Progesterone treatment significantly reduced the CBF within 30 minutes in wildtype mice at all tested concentrations tested (Anova p = 0.031; Dunnett’s post Hoc test relative to control p = 0.040, 0.041 and 0.016 for 10, 30 and 100 nM respectively; Figure 1). The average reductions in CBF were 2.10 Hz (10 nM), 1.7 Hz (30 nM) and 1.95 Hz (100 nM). Treatment with the more specific PR agonist R5020 at a concentration of 100 nM similarly reduced the CBF by 3.12 Hz (p = 0.02; Figure 2).

**Figure 3:**
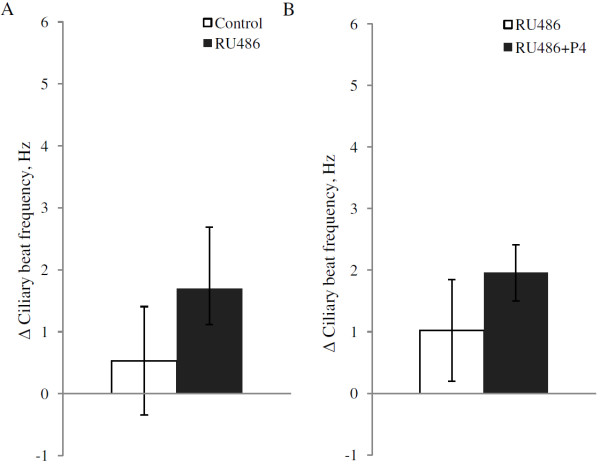
**Average change in ciliary beat frequency (CBF) for ciliary cells in mice fallopian tube after exposure to RU486.** Panel 3**A** shows cells treated with 1 μM RU486, compared to control cells treated with ethanol (EtOH). Panel 3**B** shows cells incubated with 1 μM RU486 for 30 minutes before addition of 100 nM progesterone in combination with 1 μM RU486, compared to incubation with RU486 only (1 μM) during the entire period. The CBF was measured during 90 minutes and the change in CBF is the difference between the average CBF during the last 30 minutes of the measurement and the average baseline CBF. CBFs are presented as mean ± SEM. The number of cells used in 3A were 7 in each group from in total 7 animals. For each animal, one cell was allocated to each treatment group. For 3B, the number of cells were 8 in each group from in total 10 animals.

To investigate if the PR antagonist RU486 affects the CBF by itself, cells were incubated with1 μM RU486 during 90 minutes while the CBF was measured. In both the cells treated with RU486 and the control cells there was a small increase over time in CBF with 1.69 Hz and 0.53 Hz respectively, but the effect of RU486 treatment was not significant (p = 0.23; Figure 3). To investigate if RU486 could block the effects of progesterone, cells were pretreated with 1 μM RU486 for 30 min before addition of 100 nM progesterone, then the CBF was measured for 60 minutes. Control cells were incubated with RU486 only. Similar to the results of the previous experiment, RU486 alone resulted in a non-significant increase in CBF with 1.02 Hz over time whereas RU486 together with progesterone increased the CBF by 1.9 Hz. There was no significant effect of added progesterone in the presence of RU486 (p = 0.41; Figure 3).

**Figure 4:**
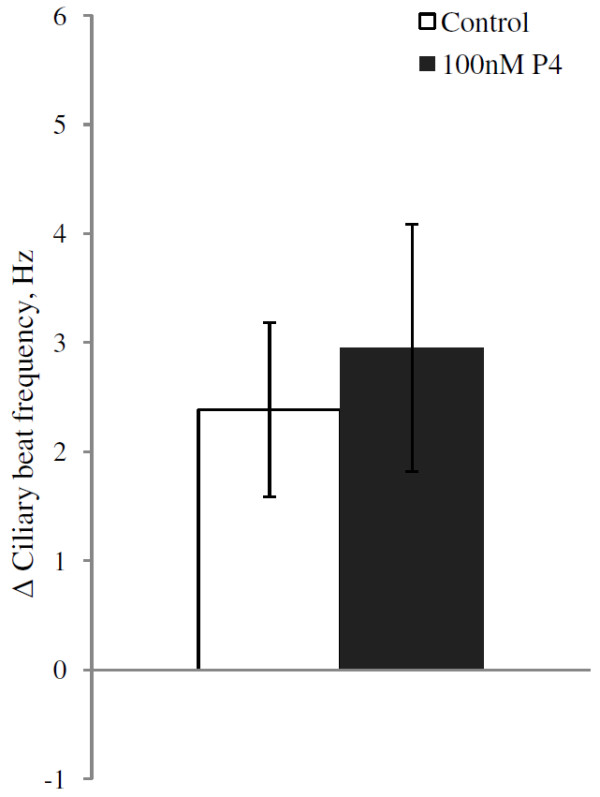
**Average change in ciliary beat frequency (CBF) for ciliary cells in the fallopian tube from mice lacking the PR, after treatment with 100 nM progesterone.** After establishment of a baseline frequency the CBF was measured during 60 minutes and the change in CBF calculated as the difference in CBF during the 30 last minutes and the baseline CBF. For each group 10 cells were used from in total 10 animals. For each animal, one cell was allocated to each treatment group.

In ciliated cells from PRlacZ mice there was no decrease in CBF after treatment with 100 nM progesterone_._ (p = 0.69; Figure 4) in contrast to the observed reduction in CBF in wildtype mice (Figure 1). The CBF increased slowly over time (3.0 Hz) and a similar (2.4 Hz) slow increase was found in control cells treated with ethanol only.

**Figure 5:**
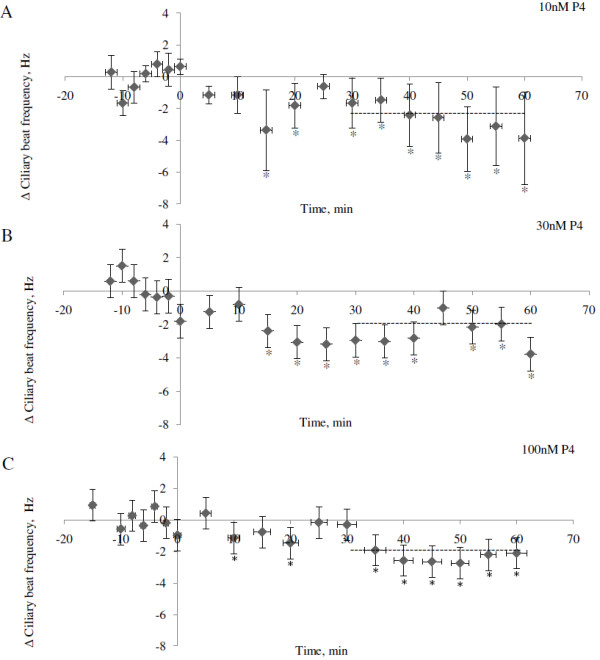
**Change in ciliary beat frequency (CBF) over time after treatment with progesterone.** CBF was measured every 5 minute for 60 minutes after addition of progesterone (**A**: 10 nM **B**: 30 nM and **C**: 100 nM) and normalized against its baseline CBF. The CBF values presented are normalized to the control cells (treated with ethanol) and expressed in relative numbers to the control groups as mean ± SEM. The drug was added at time point zero and the mean change in CBF after 30 minutes are indicated by crosshatched lines. The time points where the change in CBF is significantly different from zero is shown as * P < 0.05.

**Figure 6:**
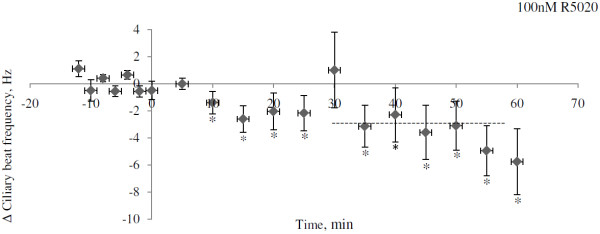
**Change in ciliary beat frequency (CBF) over time after exposure to 100 nM R5020.** A baseline frequency was established then R5020 was added and the CBF measured every 5 minutes during 60 minutes. The drug was added at time point zero and the mean change in CBF after 30 minutes are shown as a crosshatched line. The CBF values were first normalized against its baseline frequency then for each mouse the exposed cell were compared to the control cell at each time point. Then the average change in CBF at all time points was calculated and expressed as mean ± SEM. The time points where the change in CBF is significantly different from zero is shown as * P < 0.05.

Figure 5(A-C) and Figure 6 illustrates the dynamics of the change in CBF over time of cells treated with different concentrations of progesterone (Figure 5) or R5020 (Figure 6). The average reduction in CBF 30–60 minutes after addition of drug, i.e. the period used for statistical comparisons, is illustrated by vertical lines, based on paired controls with tissue from the same mice. The reduction in CBF seems to start already within 10 minutes after addition of both progesterone and R5020.

## Discussion

To investigate the possible role of PR in regulating rapid effects on tubal CBF, we studied the effects on wildtype mice of low concentrations of progesterone, a specific agonist for the PR as well as a PR receptor antagonist. We also investigated the effect of progesterone on mice lacking both isoforms of the PR. All our results support the hypothesis that the PR mediates the rapid response of progesterone on CBF in the fallopian tube.

An involvement of the PR in the long-term regulation of CBF has previously been suggested [6,8,36]. For example Nakahari et al. 2011 injected female guinea pigs subcutaneously with progesterone three times over 1.5 days and the guinea pigs were killed 13 hours after the last injection. Progesterone treatment *in vivo* decreased the CBF and this reduction was blocked if the guinea pigs were injected with RU486 [8]. As the animals were injected *in vivo*, and the response studied 1.5 days after the initial treatment, it is not possible to deduce if the regulation was direct (i.e. via PR within the fallopian tube) or indirect via stimulation of PR in other tissues. In another study Mahmood et al. investigated the effects of progesterone on human fallopian tubes *in vitro* and found that progesterone reduced the CBF with 40-50% 24 h after administration [6]. This suggests a direct effect of progesterone on cells within the fallopian tube. Neither of these studies, however, studied rapid responses, in the time frame required if the cilia are to respond and adjust their beating according to locally excreted progesterone from the travelling cumulus complex. The present study shows that within 10 minutes after administration of low levels of progesterone, there is a decrease in CBF, and this decrease is blocked if the ciliated cells are pretreated with RU486, an antagonist for the PR. Furthermore, an important piece of evidence, not presented earlier, is the lack of response in PR knockout mice. This strongly support the specificity of the action found in the wildtype mice, i.e. that the main effect of the agonists and the antagonist is indeed through the PR and not via some other receptor, such as the mPRs. Supportive of a role of the PR and not the mPRs was furthermore our finding that R5020, which bind the PR but not mPRs, gave effects similar to progesterone. Taken together, this is the first study that provides comprehensive evidence for a role of the PR in the rapid regulation of CBF in the fallopian tube of any mammal.

In a study by Wessel et al. 2004, progesterone reduced CBF 15 minutes after administration *in vitro* to cow fallopian tubes, but in contrast to our study, the effect of progesterone was not reversed by RU486 [7], leading the authors to suggest the involvement of another receptor. Importantly, in their study, 20 μM of both progesterone and RU486 was used, and it is quite possible that equimolar concentrations of antagonist did not provide sufficient competition. Furthermore, the concentration of progesterone used was very high compared to the maximum serum levels of progesterone (100 nM) in the lutheal phase found in normally cycling women [6] and in relation to the binding affinity of progesterone to PR, which is in the nM range (the k_d_ 1-5 nM [15]), thus raising questions about specificity. The same argument may be also raised regarding the study by Mahmood et al. [6]. Indeed, high non-physiological concentrations of steroids can lead to stimulation of several receptors or have non-specific effects on perturbation of cell membranes [37,38]. For example progesterone is a fairly potent agonist for the androgen receptor, and at higher concentrations, progesterone is also reported to act receptor-independently with membrane vesicles, leading to decrease membrane fluidity, induced aggregation of these vesicles and renders cells permeable to hydrophilic molecules [39]. On the other hand, the local concentration of progesterone in the follicular fluid may is considerably higher than the serum levels. In the peritoneal fluid of women the concentration of progesterone one week after ovulation reached over 900 nM [40,41]. Thus, the ciliated cells in the fallopian tube are exposed to rather high levels of progesterone during the post-ovulatory phase, perhaps particularly so in the mouse where the ovary and the fallopian tube both are located within a common fluid-filled bursa. Hence, it is still an open question what concentration range of progesterone that is physiologically relevant here. Nevertheless, we show here that 10 nM is sufficient to affect the CBF, and this is by no means an unlikely physiological concentration.

The exact localization of the PR in fallopian tube has been debated. There is immunohistochemistry data suggesting that both in the airways and in the oviduct, the PR is located to the lower half of the cilia rather than in the nucleus of the ciliated cell [13,42]. The specificity of the unusual staining in the airways is supported by the movement of the PR-staining to the nucleus after progesterone administration. On the other hand, there are also reports on immunoreactivity to PR-antibodies in the nuclear compartments of luminal epithelial, stromal, and smooth muscle cells in the mouse fallopian tube [43]. More research is apparently needed to clarify the localization of PR in the fallopian tube.

In ciliated cells of the airways, progesterone reduces the CBF with maximum reduction found 24 hours after treatment a response that is evidently genomic in nature [42]. This conclusion is based on a much slower response, a clear translocation of the PR to the nucleus after progesterone administration, and that both the translocation and the effect on CBF could be blocked by the antagonist for PR RU486. Furthermore, the CBF was also blocked by the transcription inhibitor, actinomycin D. A puzzling observation with the study on airways [42], also acknowledged by the authors, is that a much higher concentration of progesterone was required (20 μM) than in the fallopian tube (10 nM) studied here.

## Conclusions

In the fallopian tube progesterone rapidly reduces the CBF via the classical PR but probably via a non-classical mechanism. The function of such a rapid response here might be to enable progesterone, secreted from the travelling cumulus complex, to locally reduce the CBF. This would in turn be expected to modulate the transport speed of the cumulus complex, in order to increase the likelihood for fertilization to take place within the ampullary region. The evidence presented in this study indicates a crucial role of PR in this process. Further studies on gene transcription and second messenger systems, as well as the use of transcription and translation inhibitors could shed further light on the detailed mechanisms involved. Such knowledge may improve our possibilities to understand the mechanisms involved in ectopic pregnancies associated with disturbed progesterone signaling, and possibly reduce the risks for such conditions to develop [44].

## Competing interests

All authors declare that they have no competing interests.

## Authors’ contributions

AB, DGJL, HB and MG designed the study. AB carried out the tissue preparations and CBF measurements, analysed the data, performed the statistical analysis and drafted the manuscript. DGJL also took part in the statistical analyses and helped to draft the manuscript. MG helped to draft the manuscript. KL helped with some of the tissue preparation and the CBF measurements. DGJL supervised the study with help from MG and HB. All authors read and approved the final manuscript.
